# Prevalence of active hepatitis c virus infection in district mansehra pakistan

**DOI:** 10.1186/1743-422X-7-334

**Published:** 2010-11-22

**Authors:** Amjad Ali, Habib Ahmad, Ijaz Ali, Sheema Khan, Gulshan Zaidi, Muhammad Idrees

**Affiliations:** 1 Department of Genetics, Hazara University Mansehra, Pakistan; 2 Department of Botany, Hazara University, Mansehra, Pakistan; 3 Institute of Biotechnology & Genetic Engineering, Agricultural University Peshawar, Pakistan; 4 Medical B-ward, Khyber Teaching Hospital Peshawar, Pakistan; 5 Division of Molecular Virology, National Centre of Excellence in Molecular Biology, 87-West Canal Bank Road Thokar Niaz Baig Lahore-53700, University of the Punjab, Lahore, Pakistan

## Abstract

Prevalence of active hepatitis C virus (HCV) infection in apparently healthy inhabitants of District Mansehra, Pakistan was surveyed during September, 2009 to May, 2010. Subjects of different age and gender groups were analyzed through random blood sampling from people of three areas viz; Tehsil Mansehra, Tehsil Balakot and Tehsil Oghi. Sum of 400 individuals, 300 male and 100 females with age groups from 10 years to 50 and above were included in the study. All the individuals were screened for antibodies against HCV. The positive samples thus screened, were subjected to polymerase chain reaction (PCR) analysis for detection of HCV-RNA. The results showed that 3.5% of the people of District Mansehra are actively infected with HCV whereas 7% of the population in general, has the presence of antibodies against HCV in their blood. It was also concluded that the prevalence of active HCV infection was high 4% in males as compared to females (2%). The prevalence of HCV proportionality increases with the increase in age of the people. Its incidence was highest (7.69%) in the people of the age group of 51 years and above, whereas no sign of infection was recorded for the age group of 10-20 years.

## 1. Background

Human hepatitis C is an infectious disease affecting the liver, caused by the Hepatitis C Virus (HCV). The infection is often asymptomatic, but once established, it can progress to the fibrosis of liver and ultimately cirrhosis. In some cases, those with cirrhosis will go on to develop liver failure and other complications including liver cancer [1]. HCV is the only known member of the *Hepacivirus *genus in the family *Flaviviridae*. It is single stranded 50 nm positive sense RNA virus with six major genotypes causing hepatitis C in the whole of the world [2,3]. It is reported that approximately 15-40% of persons infected with HCV clear the virus from their bodies during the acute phase of infection and the remaining 60-85% of patients infected with HCV develops chronic hepatitis C [4], which progresses to liver cirrhosis with an elevated risk of the development of hepatocellular carcinoma [2,5,6].

There are about 170 million patients with HCV in the world and three to four million individuals are diagnosed as new cases every year [7,8]. Pakistan, a developing nation of 170 million people has alarmingly rate of outbreaks of hepatitis C virus [9-12] which need proper survey and genotyping. Seroprevalenc studies of anti-HCV antibodies in the general population of Pakistan have been recorded as 5.31% to 7.5% [13-15]. HCV prevalence is in the range of 4.1 to 36% reported from various parts of Khyber Pukhtoonkhwa Province of Pakistan [16,17] but no data has been reported till now on the prevalence of HCV from District Mansehra.

Majority of the studies conducted have focused on the prevalence of anti-HCV antibodies which is least informative about the active HCV infection. PCR has emerged as a powerful molecular diagnostic tool for the detection of active infection which is manifested by the presence of HCV RNA in the blood of the infected person. As no study has earlier been conducted to figure out the prevalence of anti-HCV antibodies or HCV RNA among the general population of District Mansehra, we for the first time conducted our study to find out prevalence of active HCV infection in general population of District Mansehra.

## 2. Methods

### Blood Sampling

The study included individuals from all over Mansehra District of Khyber Pakhtunkhwa province. Informed consent was taken from individuals under observation. A total of 400 apparently healthy individuals comprised of 300 males and 100 females of different age groups were included in the study. History of volunteers was recorded in the form of questionnaires.

Random blood sampling was done from the three Tehsils of District Mansehra, namely Tehsil Mansehra, Tehsil Balakot and Tehsil Oghi. From every volunteer 5 ml of blood was collected in separate disposable sterile syringes. Blood was transported to Institute of Biotechnology & Genetic Engineering (IBGE), Peshawar where it was centrifuged for 5 minutes at 15000 rpm to separate serum.

### Immuno-chromatographic tests (ICT)

Sera screening was done for anti-HCV antibodies with the help of Immuno-chromatographic tests by using strips from (Accurate, USA) followed by (Acon, USA). The positive samples were subjected to further analysis.

### RNA Extraction and PCR

HCV RNA was extracted from 200 μl serum sample by using Ana-gen RNA extraction kit (Ana-gen, USA) according to manufactures' instructions. cDNA was prepared by Reverse transcription PCR using M-MLV reverse transcriptase (Fermentas, USA). The amplified cDNA was further subjected to two rounds of PCR amplifications using nested primers [18]. The conditions for the first round PCR were as follows; An initial denaturation step at 95°C for 2 minutes followed by 30 cycles of 94°C for 45 seconds, 54°C for 45 seconds, and 72°C for 1 minute performed in a thermal cycler (Eppendorf, Germany). The conditions for the 2nd round PCR were the same except that a different set of inner primers was used and the annealing temperature was raised to 62°C in order to amplify the 1st round product.

### Gel Electrophoresis and Documentation

All the PCR products (first and second rounds) were analyzed on 1.8% agarose gel prepared in 0.5% TBE buffer, stained with ethedium bromide (10 mg/ml) as florescent dye. Gels were photographed using Alpha quant (Alpha Innotech). A 100-bp DNA ladder (Gibco BRL) was used as DNA size marker.

### Statistical Analysis

SPSS version 14.0 for windows was used for the statistical analysis of the data. The results were obtained in rates (%).

## 3. Results

A total of 400 apparently healthy individuals were randomly sampled for the study. Theses individuals belonged to various Tehsils of District Mansehra namely Tehsil Mansehra, Tehsil Balakot and Tehsil Oghi. Out of the total 400 samples examined, 300 were males while 100 samples were from the female population (table 1).

**Table 1:** Basic information about the blood samples collected from District Mansehra

Tehsil	No. of samples	Male	Female
**Mansehra**	170	120	50

**Balakot**	130	98 act	32

**Oghi**	100	82	18

			

**Total**	400	300	100

All the individuals were categorized into five age groups. Sera were isolated from all the blood samples and subsequently tested for anti-HCV antibodies by Immuno Chromatographic test (ICT). The samples were first tested using ICT strips from Accurate (USA), followed by ICT strips from Acon (USA). The results indicated that 28 out of 400 individuals had anti-HCV antibodies in their blood. These individuals belonged to various age groups (Table 2). As anti-HCV antibodies are not informative about the active HCV infection therefore all the anti-HCV positive samples were processed for RNA extraction and RT PCR.

**Table 2:** Age wise distribution of the anti- HCV antibodies and HCV RNA

Age group	No. of samples	Anti-HCV +ve	HCV RNA +ve
**10-20 years**	48	0 (0%)	0 (0%)

**21-30 years**	110	4 (3.64%)	2 (1.82%)

**31-40 years**	94	6 (6.38%)	2 (2.13%)

**41-50 years**	70	10 (14.28%)	4 (5.71%)

**51 and above**	78	8 (10.26%)	6 (7.69%)

			

**Total**	400	28 (7%)	14 (3.5%)

**Figure 1:**
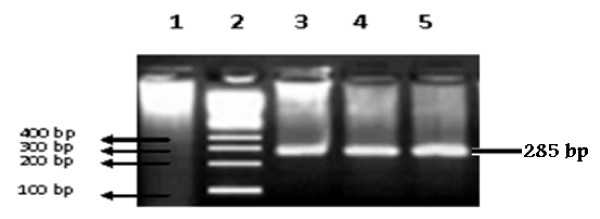
**Typical gel photograph of HCV amplified products**. Lane 1 is negative control, lane 2 indicates 100 bp DNA size marker used in the study, lane 3, 4 and 5 are the positive samples for active HCV which show 285 bp band of hepatitis C virus.

Anti- HCV positive samples were subjected to PCR. The PCR results revealed that 14 (3.5%) individuals had active HCV infection as indicated by the detection of HCV RNA in their blood (Figure 1).

**Figure 2:**
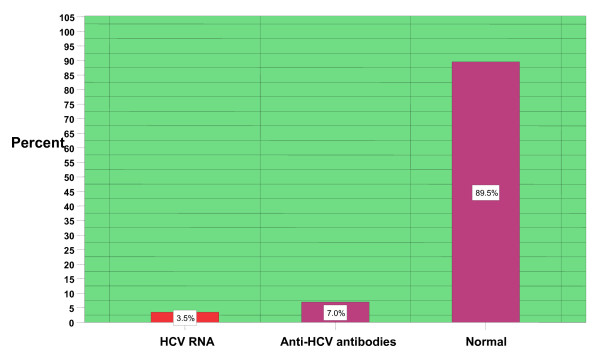
**Distribution of HCV in District Mansehra, Pakistan**. The active infection is 3.5% as described in first bar. The second bar indicates that 7% percent of the people of District Mansehra carry anti- HCV antibodies in their blood. The last bar indicates the normal population of District Mansehra which is 89.5%.

The prevalence of anti-HCV antibodies and HCV RNA in the case of male and female population as determined by the combination of Immuno Chromatography procedures and PCR is given in Table 3. Over all result revealed that active HCV infection in District Manehra is 3.5% (Figure 2).

**Table 3:** Sex- wise distribution of the anti- HCV antibodies and HCV RNA

Sex	Total No. of samples	Anti-HCV +ve	HCV RNA +ve
**Male**	300	22 (7.33%)	12 (4%)

**Female**	100	6 (6%)	2 (2%)

			

**Total**	400	28 (7%)	14 (3.5%)

## 4. Discussion

Hepatitis C is rapidly emerging as a major health problem in developing countries including Pakistan [19,20]. The World Health Organization estimates that approximately 3% of the world populations has been infected with HCV thus far [21]. There are about 170 million patients with HCV in the world and three to four million individuals are diagnosed as new cases every year [22,23].

Prevalence of HCV may be different in different regions and various groups of the same community [24]. Hospital-based studies revealed prevalence rates of HCV as 5.31% in Islamabad [25], 2.45% -20.89% in various parts of the Punjab province [26-28], 4-6% in Karachi [29], 5%-9% in North West Frontier Province (N-W.F.P) [30,31] and 25.7% in Northern Areas [32]. Slightly higher prevalence of HCV was recorded in the earth quake affected areas of Pakistan in 2005 [20].

For the investigation of the active HCV infection we relied on the highly sensitive method of detection that is Polymerase Chain Reaction. We collected 400 hundred samples from the general apparently healthy male and female populations of the District Mansehra, Pakistan and subjected them to ICT and PCR. 3.5% individuals turned out to be actively infected with HCV. Anti-HCV antibodies were found in 7% of individuals. Higher percentage prevalence of anti-HCV antibodies could be attributed to the false positive results as are experienced during Immuno- Chromatographic strip Tests or the self limiting nature of the HCV infection.

The study revealed that young people of district Mansehra (in the age groups of 10-20 years) have no active HCV infection. The absence of HCV infection in this age group may be due to their least exposure to some of the high risk factors causing HCV such as exposure to barbers etc. The highest active HCV infection was 7.69% observed in age group 51-60. These people had variable history of exposure to HCV risk factors such as major/dental surgery or blood transfusion. Second highest active HCV infection was 5.71% observed in age group 41-50. Our study is partially in agreement with one study [31], which was conducted on the prevalence of HCV in relation to age groups and observed that High prevalence of HCV was among middle-aged (40-50 years) people. Third highest active HCV infection was 2.13% observed in age group 31-40 years. People including in age group 21-30 years revealed 1.82% active HCV infection. High prevalence of HCV infection in male population has earlier been recorded by other studies from Pakistan and around the globe. The prevalence of HCV in different age groups of both sexes was studied and it was found that the prevalence of HCV was maximum (8.92%) in mature males as compared to young males (6.66%) [33]. Our study is in agreement with the fact that male population is more affected by HCV as is indicated by HCV prevalence of 4% in the male population of District Mansehra. The female population is least affected with 2% active prevalence as compared to male population of District Mansehra. High prevalence in male and lower prevalence in female again could be attributed to their exposure status to various HCV risk factors which was quite evident from the life style and history of the individuals sampled for this study. Over all our study reveals that 7% of the population of district Mansehra have antibodies against HCV in their blood while 3.5% people are actively infected with HCV.

## Conclusion

The prevalence of active HCV recorded in different age and gender groups show that its frequency has increased with the increase in age. Children whether males or females were the least infected whereas its prevalence was highest in the age group of 51 years and above. Furthermore the rate of infection in female population was almost 50% as compared to males. The least incidence of HCV in females could be attributed to low exposure to HCV risk factors due to male dominating society of the area and also the estrogen hormone in females is considered to play a role in the spontaneous clearance of HCV infection [34].

## Abbreviations

cDNA: complementary DNA; HCV: hepatitis C virus; IBGE: Institute of Biotechnology and Genetic Engineering; ICT: Immno-chromatographic Test; KP: Khyber Pukhtoonkhwa; M-MLV: Molony-murine leukemia virus; PCR: Polymerase chain reaction; RNA: Ribonucleic acid; RT PCR: Reverse transcription polymerase chain reaction; TBE: Tris, Borate, EDTA.

## Competing interests

The authors declare that they have no competing interests.

## Authors' contributions

HA conceived the study, participated in its design and coordination and gave a critical view of manuscript writing. AA collected the prevalence data, performed screening, determined active HCV and analyzed the data statistically. IA helped AA in molecular assays and gave a critical view of manuscript writing and participated in data analysis. MI critically reviewed the manuscript. Remaining authors helped in collection of the data from various regions of the District Mansehra. All the authors read and approved the final manuscript.
